# RAF Inhibitor Therapy Promotes Melanocytic Antigen Expression and Enhanced Anti-Tumor Immunity in Melanoma

**DOI:** 10.4172/2376-0427.1000139

**Published:** 2014-10-16

**Authors:** Alexandre Reuben, Rodabe N Amaria, Zachary A Cooper, Jennifer A Wargo

**Affiliations:** 1 Division of Surgical Oncology, The University of Texas MD Anderson Cancer Center, 1515 Holcombe Blvd, Houston, TX 77030, USA; 2 Melanoma Medical Oncology, The University of Texas MD Anderson Cancer Center, 1515 Holcombe Blvd, Houston, TX 77030, USA; 3 Department of Genomic Medicine, The University of Texas MD Anderson Cancer Center, 1515 Holcombe Blvd, Houston, TX 77030, USA

**Keywords:** Melanoma, BRAF, Targeted therapy, Cancer, T cells

## Abstract

Melanoma remains a major cause of morbidity and mortality worldwide, however tremendous advances have been made in its treatment over the past several years. The discovery of genomic alterations that contribute to oncogenicity has ushered in a new era of molecularly-targeted therapy. Importantly, over half of melanomas harbor a mutation in the BRAF gene that leads to constitutive signaling down the MAPK pathway and multiple subsequent deleterious effects. Pharmacologic agents targeting this mutation have been developed and several are now FDA-approved, having yielded high response rates to therapy although these are tempered by a short duration of response. Multiple molecular mechanisms of resistance have been identified; however until recently few studies had delved into the immune effects of BRAF inhibitors. The effect of BRAF inhibition on anti-tumor immunity will be discussed herein, as will potential implications of these findings in the treatment of melanoma.

## Introduction

There have been major advances in the treatment of melanoma in recent years, however late stage disease remains a fatal diagnosis for the majority of patients. Moreover, the incidence of melanoma is increasing at an alarming rate; faster than any other solid tumor [1,2]. Over the years, multiple oncogenic mutations have been identified in melanomas, and drugs targeting these mutations have been developed. The most common mutation is in the BRAF gene, which drives constitutive signaling of the MAPK pathway with multiple deleterious effects [3,4]. Mutations in the BRAF gene occur in approximately 50% of melanomas [3,5], and multiple agents are now FDA-approved for the treatment of patients with BRAF-mutant metastatic melanoma (vemurafenib, dabrafenib and trametinib). Treatment of patients with the BRAF specific inhibitor vemurafenib yields impressive results including an overall response rate of 53% (47% with a partial response and 6% with a complete response) [6]. However, responses to BRAF inhibitor monotherapy are not durable with an increase in progression free survival of 6.8 months and a median overall survival of 16 months [6]. To combat resistance, investigators proposed targeting two nodes in the same pathway by treating with inhibitors to BRAF^V600E^ and MEK. Treating patients with this regimen extended progression-free survival to 9.8 months but resistance to therapy remains an issue [7].

Another potential way to improve responses to BRAF-targeted therapy may be by combining it with immunotherapy [8,9]. This concept is based on the fact that BRAF inhibitors (BRAFi) have been shown to significantly affect anti-tumor immunity, essentially creating a more favorable tumor microenvironment early during the course of therapy [9–11]. This review specifically addresses the effects of BRAF-targeted therapy on melanocyte antigen expression and on the tumor microenvironment, and discusses implications of this data.

## Expression of Melanocyte Antigens

A key step required for T cell cytotoxicity of tumors is their capacity to recognize their cognate antigen. However, tumors often develop mechanisms to escape antigen presentation, through down-regulation of the expression of antigens directly [12,13], or through alterations of the major histocompatibility complex (MHC) class I and II components required for proper surface expression of antigens [14,15]. Because T cells are dependent on the presence of cognate antigen in order to respond to and lyse malignant cells, their capacity to destroy tumor cells may be impeded even when they do infiltrate tumors due to tumor antigen escape.

**Figure 1:**
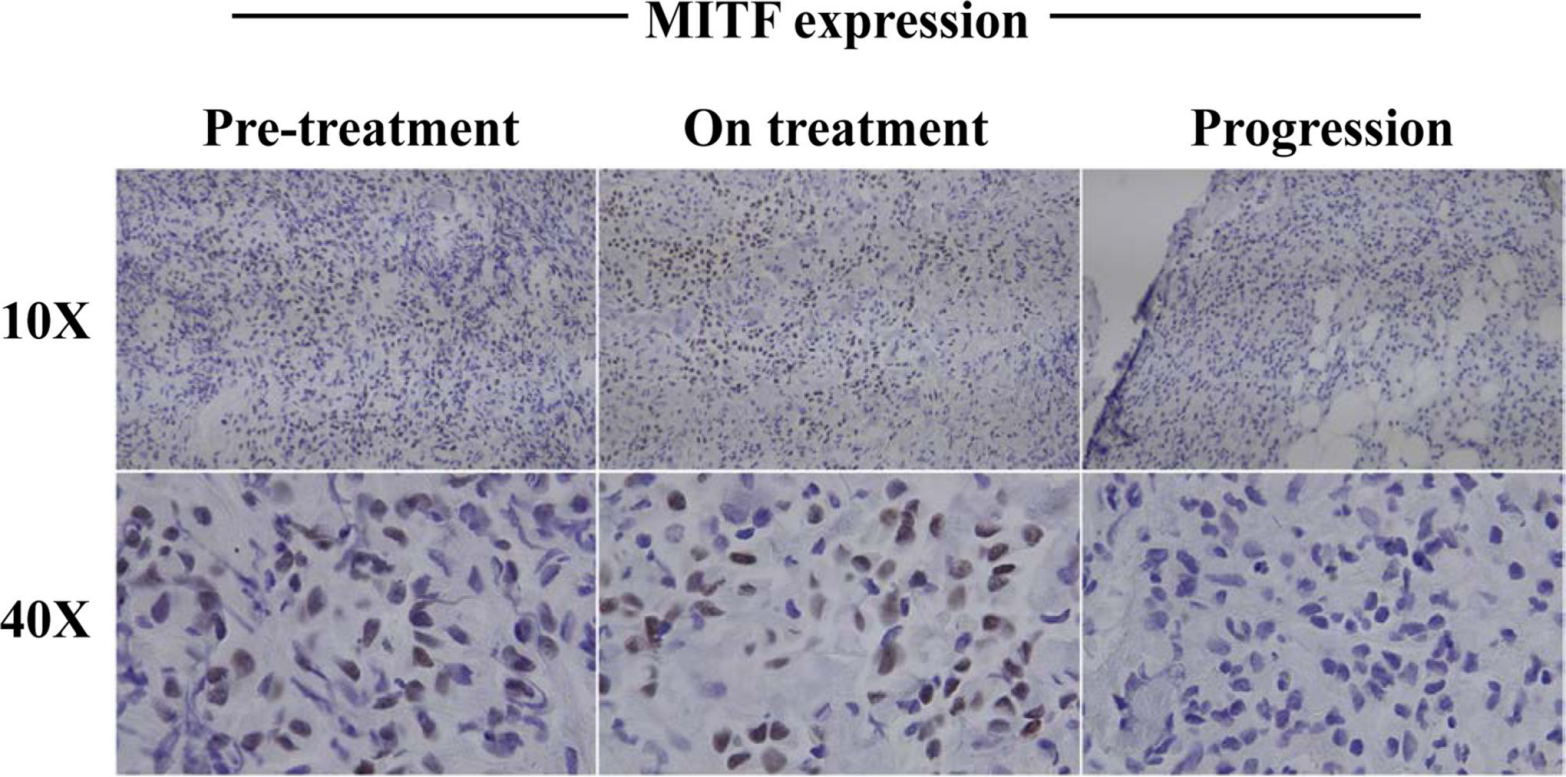
Induction of melanocyte antigen expression upon BRAFi treatment and loss at progression. Representative staining for MITF expression in a patient with metastatic melanoma at 10X and 40X shows an increase upon initiation of BRAFi and a decrease at disease progression on therapy.

Interestingly, treatment with BRAF inhibitors has demonstrated surprising effects on tumor antigen expression in the context of melanoma. This was first studied *in vitro*, where treatment of BRAF-mutant melanoma cell lines was associated with a significant increase in expression of Melanocyte Differentiation Antigens (MDA), including MART-1, gp-100, TYRP-1 and TYRP-2. Importantly, this increase in antigen expression was associated with enhanced reactivity of antigen-specific T cells [16]. This concept was next studied in patients with BRAF-mutant metastatic melanoma by performing pre-treatment (day 0) and on-treatment (day 10–14) tumor biopsies. MDA expression was assayed via quantitative Polymerase Chain Reaction (qPCR) and Immunohistochemistry (IHC), and demonstrated a significant increase in MDA expression across the board, as well as an induction of Microphthalmia-Associated Transcription Factor (MITF), a transcription factor regulating their expression (Figure 1) [10]. Upon disease progression, antigen expression was lost, however, and even dropped below pre-treatment levels [10]. Therefore, MITF induction by BRAFi may result in better expression and presentation of MDA, thereby favoring tumor cell recognition and clearance by patrolling T cells. Furthermore, antigen escape by tumors may be more readily overcome if multiple antigens are induced rather than one sole antigen that may more easily be mutated away from immunogenicity.

MHC class I and II molecules are responsible for proper antigenic presentation of immunogenic peptides. Accordingly, the chaperones and components regulating these pathways are often targeted by tumors to escape the tumor surveillance enforced by CD8^+^ and CD4^+^ T lymphocytes. Antigenic recognition is impossible in absence of MHC molecules, and so it comes as no surprise that melanomas may be selected for their loss of MHC I expression [13]. In melanoma, it was observed that MHC I expression is down-regulated in BRAF^V600E^-mutated cells, potentially due to increased internalization of surface MHC I molecules [17]. However, treatment with a BRAFi resulted in restored expression of MHC I molecules at the cell surface. Furthermore, Sapkota and colleagues demonstrated that the BRAFi vemurafenib enhances MHC I induction in human melanoma cells [15]. Interestingly, BRAFi treatment enhanced both MHC I and MHC II induction by interferon gamma (IFN-γ), through increases in CIITA and NLRC5 expression [15]. These results suggest that not only are MDA increasingly expressed upon BRAFi treatment, but they also may be better recognized by patrolling T cells due to increases in MHC I and MHC II proteins which act as adaptors between antigen and T cell.

## T Cell Infiltrate Following BRAF Inhibition

The proper killing of tumor cells by the immune system requires the presence of T cells in the immediate environment which is a benefit lacking in the majority of tumor hosts. Tumors deploy multiple mechanisms in order to ensure the improper recruitment and accumulation of T cells at the tumor site by induction of ligands such as Fas-ligand [18] as well as the production of vascular endothelial growth factor (VEGF) [19] and immunosuppressive cytokines such as interleukin (IL)-6 and IL-8 [10]. There is evidence that oncogenic BRAF is immunosuppressive [20], and T cell infiltrates in BRAF-mutant melanomas are low prior to initiating therapy with a BRAFi [10]. However, following the administration of a BRAFi in patients with melanoma, tumor biopsies demonstrated a higher infiltration of both CD4^+^ and CD8^+^ T lymphocytes within 2 weeks of treatment initiation [10,21]. Importantly, this was associated with a decrease in immunosuppressive cytokines and VEGF in the tumor microenvironment [9–11]. Further studies have pursued the effect of BRAFi on chemokine, cytokine and growth factor levels in the serum. Wilmott et al. demonstrated significant increases in IFN-γ, CCL4 and tumor necrosis factor-α and a decrease in IL-8 in early on-treatment serum samples which significantly correlated with a decrease in Ki67 and an increase in CD8^+^ T cell density within the tumor [22]. Increased T cell recruitment to tumors induced by BRAFi treatment may promote the sensitivity of tumors to cytotoxic T cells.

## Effects of BRAFi on the Expression of Immunomodulatory Molecules

Recent studies in the treatment of melanoma have confirmed the importance of immunomodulatory molecules, specifically the programmed death-1 (PD-1) pathway, on anti-tumor activity. PD-1 is expressed on activated T and B cells [23] while its major ligand, programmed death ligand-1 (PD-L1), may be expressed on a certain class of macrophages and can be induced by inflammatory cytokines in various tissue types [24–28]. T cell function is repressed when T cells expressing PD-1 bind to PD-L1 [25]. In a recent study, 38% of 150 melanocytic lesions stained positive for PD-L1 [28]. Interestingly, 10–14 days after metastatic melanoma patients were treated with BRAFi a significant upregulation of PD-1 and PD-L1 was demonstrated [10]. Additionally, Jiang et al. showed that melanoma cells resistant to BRAFi showed an increase in MAPK signaling and in PD-L1 expression [29]. In contrast, the upregulation of PD-L1 that accompanied melanoma cell line resistance to vemurafenib was linked to the activation of alternative signaling pathways rather than reactivation of the MAPK pathway [30]. Combined, these findings suggest that PD-L1 may contribute to a mechanism of resistance to BRAF-targeted therapy. Additional immunomodulatory molecules such as TIM-3, LAG-3, CTLA-4, BTLA and others may also play a role in response and resistance to BRAF-targeted therapy and must be further studied.

## Effects of BRAF and MEK Inhibition on T Cell Reactivity

In addition to enhancing reactivity to antigen-specific T cells through increased MDA expression, BRAFi may also have a direct effect on T lymphocytes. This was first studied *in vitro* by Boni et al, who demonstrated that treatment of T lymphocytes with a BRAFi had no deleterious effects on T cell proliferation and function, whereas treatment with a MEK inhibitor did [16]. This is highly relevant, as T cells rely heavily on the MAPK pathway for activation. This work was complemented and enhanced by that of Callahan et al, who demonstrated that treatment of T lymphocytes with BRAFi led to paradoxical activation and increased signaling through ERK [31]. This has important implications, as BRAFi may have a two-pronged impact on tumor destruction, by both sensitizing tumor cells to apoptosis, and maintaining the capacity of T lymphocytes to infiltrate and destroy tumor cells.

The clinical implications and effect of MEK inhibition on T cells in patients with metastatic melanoma is unclear. Though *in vitro* studies suggested a deleterious effect [16], there was no difference in T cell infiltrate in tumor biopsies of patients treated with BRAF inhibitor monotherapy versus therapy with combined BRAF and MEK inhibitors [10]. Further *in vitro* studies by Vella et al. suggest that MEK inhibition alone or in combination with BRAFi may affect T lymphocyte proliferation, cytokine production and antigen-specific expansion [32]. This concept is being actively studied in the context of human clinical trials, and insights gained will be relevant in the treatment of melanoma as well as other cancers.

## Antigen Specificity of the T Cell Response

A critical question with regard to the T cell infiltrate observed in the setting of BRAFi is whether it is of antigen-specific nature. T cell populations expand from a single clone, which recognizes a cognate antigen. Therefore, depending on the antigens present, certain T cell clones may expand and contract upon clearance whereas others may remain unaffected. As mentioned, treatment with BRAFi in patients with metastatic melanoma is associated with an increased T cell infiltrate [10], though it is unclear if this is an antigen-specific response, or whether T cells infiltrate the tumor mass following significant tumor necrosis. Tumor biopsies obtained in these patients are relatively small, thus an exhaustive analysis of antigen specificity by flow cytometry and tetramer analysis or ELISPOT is technically not feasible in most cases. However, some insight has been gained through the use of T cell receptor sequencing in the setting of BRAFi treatment, suggesting that this is more likely related to an antigen-specific response [33]. In these studies, a more clonal T cell population was found in patient tumor samples following 2 weeks on a BRAFi. Interestingly, the majority of clones in these on-treatment tumors were new, suggesting infiltration of the tumor rather than proliferation of pre-existing clones. Furthermore, there was an association between the T cell repertoire and response, demonstrating that response may be associated with pre-existing T cell clones [33]. This data does not suggest that the response is specific to melanocyte antigens, and this is still an important question, particularly in light of the recent evidence for neoantigens mediating responses to anti-cancer therapy [34,35].

## Proposed Model for the Effects of BRAFi on Anti-Tumor Immunity

**Figure 2:**
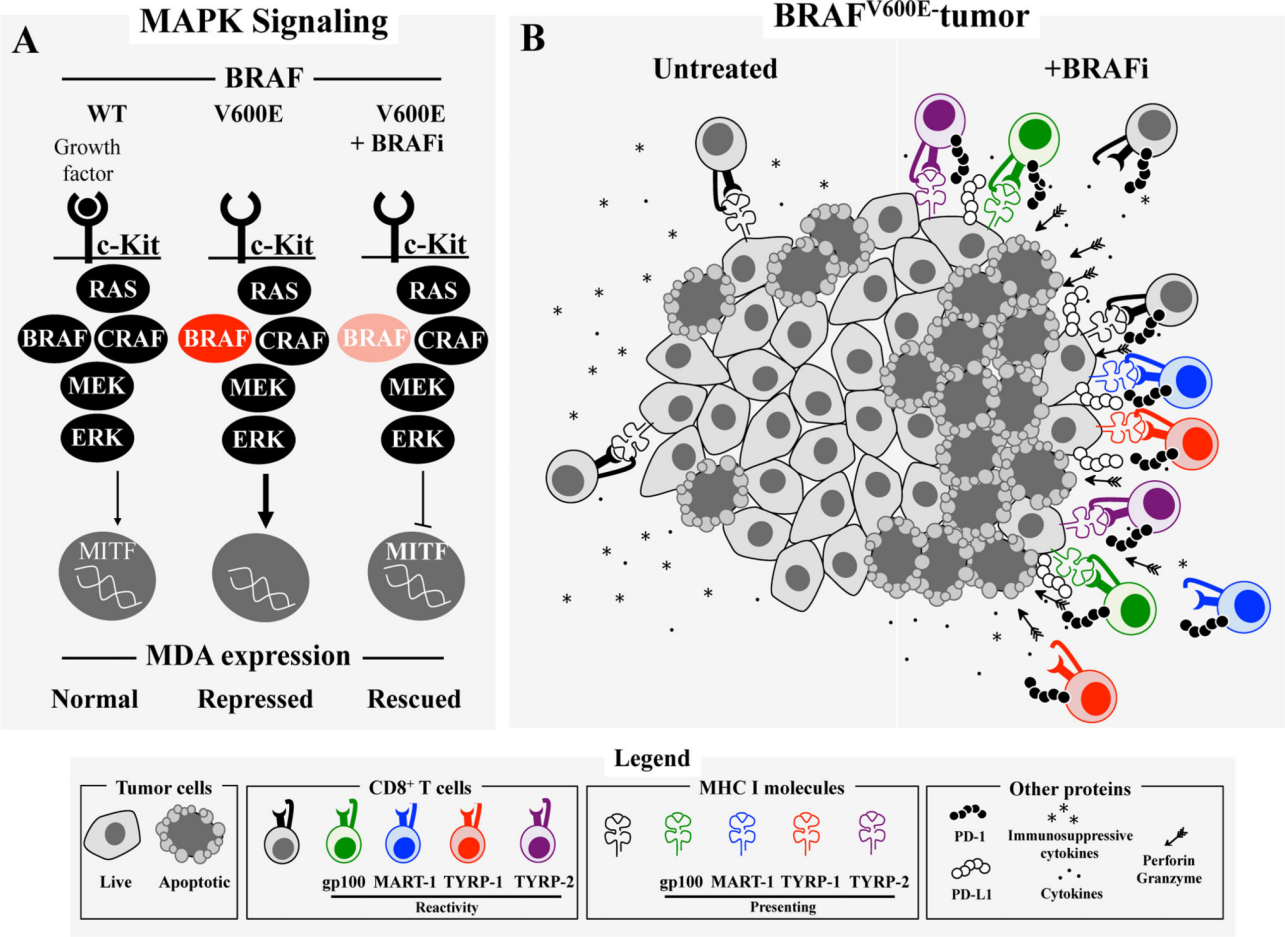
Overview of impact of BRAFi on T cell response to melanomas. A) Summary of MAPK signaling pathway and downstream effects on MITF and melanocyte differentiation antigen (MDA) expression. Constitutive BRAF signaling caused by BRAF^V600E^ results in inhibition of MITF and downstream MDA expression whereas BRAFi rescues MITF and subsequent MDA expression. B) Overview of the immunosuppressive microenvironment in BRAF-mutant melanoma and of the immune-based antitumor effect after initiation of a BRAFi including an increase in T cells, melanoma cell death, cytokines, perforin, granzyme B, MDA, PD-1, PD-L1 and a decrease in immunosuppressive cytokines.

Based on the available data, we propose the following model for the effects of BRAFi on anti-tumor immunity (Figure 2). First, the oncogenic BRAF mutation contributes to immune escape in melanoma tumors by transcriptional repression of MITF and low MDA expression [10,16,36]. This is further potentiated by down-regulation of MHC I [17]. In addition, the tumor microenvironment secretes high levels of immunosuppressive cytokines and VEGF [9–11]. Treatment with a BRAFi results in a release of the transcriptional repression of MITF, thus allowing for increased expression of MDA [10], which are then processed and presented on the surface of the cell in the context of MHC molecules which are increasingly induced by IFN-γ following BRAFi therapy [17]. The production of immunosuppressive cytokines and VEGF are also reduced while an increase in cytotoxic factors such as granzyme B and perforin are seen in the setting of treatment [9,10]. Together, these effects promote infiltration of T cells into the tumor as well as clonal expansion of pre-existing T cells, though the antigen specificity of this response is still unclear.

## Summary and Implications for Treatment

Melanoma remains a major cause of morbidity and mortality worldwide, yet significant advances have been made in treatment through the use of targeted therapy and immunotherapy. There is growing evidence that treatment with targeted therapy (namely BRAFi) has a positive effect on the immune system early during the course of treatment which may contribute to the responses observed. However simultaneously there is an increase in the expression of immunomodulatory molecules on the surface of T cells and in the tumor microenvironment. These results have important implications, though several questions remain. The kinetics of the immune response to BRAFi are not clearly defined, though this is an area of intense investigation. This has critical translational relevance, as there may be synergy when combining treatment with targeted therapy and immunotherapy [8,9,37] though the optimal timing for adding immunotherapy to a backbone of targeted therapy remains unknown. In addition, the impact of MEK inhibitors (either as monotherapy or in combination with BRAFi) is poorly understood. Ultimately, ideal approaches using combined BRAF-targeted therapy and immunotherapy for the treatment of melanoma will be built on a deep understanding of the molecular and immune effects of each of these therapies in isolation, as well as in combination.
